# Targeted Nanoliposomes for the Delivery of Boronophenylalanine into HER2-Positive Cells

**DOI:** 10.32607/actanaturae.27722

**Published:** 2025

**Authors:** G. M. Proshkina, E. I. Shramova, A. B. Mirkasymov, I. N. Zavestovskaya, S. M. Deyev

**Affiliations:** State Research Center “Shemyakin–Ovchinnikov Institute of Bioorganic Chemistry RAS”, Moscow, 117997 Russia; Lebedev Physical Institute RAS, Moscow, 119991 Russia; National Research Center “Kurchatov Institute”, Moscow, 123098 Russia; Moscow Institute of Engineering Physics, National Research Nuclear University “MEPhI”, Moscow, 115409 Russia; Ogarev National Research Mordovia State University, Saransk, 430005 Russia

**Keywords:** boron neutron capture therapy, HER2-specific therapy, liposomes, 4-L-10BPA

## Abstract

Boron neutron capture therapy (BNCT) is a rapidly developing field of radiation therapy for cancer that is based on the accumulation of the radiosensitive 10B isotope in cancer cells, followed by tumor irradiation with thermal neutrons. Widespread use of BNCT in clinical practice remains limited because of the poor accumulation of boron-containing (10B) drugs in the tumor or their high toxicity to the body. This study focuses on the engineering of tumor-specific liposomes loaded with 4-L-boronophenylalanine (4-L-10BPA) for application in boron neutron capture therapy. According to the spectrophotometry and ICP-mass spectroscopy data, the 4-L-10BPA-to-liposome molar ratio is ~ 120,000. Liposomal targeting of human epidermal growth factor receptor 2 (HER2) was determined by HER2-specific designed ankyrin repeat protein (DARPin)_9-29 on the outer surface of liposomes. DARPin-modified liposomes were found to bind to HER2-overexpressing cells and be effectively internalized into the cytoplasm. The ability of DARPin-functionalized liposomes to precision-deliver large quantities of 4-L-10BPA into cancer cells may open up new prospects for BNCT.

## INTRODUCTION

Boron neutron capture therapy (BNCT) is a method used to treat malignant tumors by which the radiosensitive ^10^B isotope preliminarily accumulated in the tumor is subjected to neutron irradiation. Neutron absorption by ^10^B is accompanied by a nuclear reaction with a substantial energy release that leads to cell death: ^10^B + ^1^n → ^4^He(α) + ^7^Li + 2.4 MeV [1]. Hypothetically, only ^10^B-loaded cells are expected to die as a result of this reaction, since alpha particles and lithium nuclei experience rapid deceleration and have short penetration ranges in biological tissues (5–9 μm); approximately equal to the diameter of a single cell. Hence, the cytotoxic effect is supposed to be confined to the immediate vicinity of the reaction site [2]. BNCT is currently under intensive development, with efforts focused on designing compact accelerator- driven neutron sources and 10B-containing agents characterized by *in vivo *biocompatibility and stability [3].

Today, a phenylalanine derivative containing a ^10^B atom, 4-borono-L-phenylalanine (4-BPA), is the only available drug approved for clinical application in BNCT. Thus, 4-L-^10^BPA has been approved for use under the trade name Borofalan (Steboronine®) as a medication for the treatment of locally recurrent head and neck cancer in Japan since 2020 [4].

The problems limiting the use of 4-L-^10^BPA are related to its low accumulation in cancer cells and poor solubility in water.

4-L-^10^BPA is delivered into cancer cells via the active transport mechanism, through L-type amino acid transporters (mainly LAT-1 [5, 6]), which is independent of both pH and the concentration of Na^+^ ions. LAT-1, a heterodimer transmembrane protein, is involved in the delivery of neutral amino acids with branched side chains (valine, leucine, and isoleucine), as well as aromatic amino acids (tryptophan and tyrosine) into the cell [7, 8, 9]. This transporter is likely to be overexpressed in many tumor types [10] and can be regarded as a target for 4-L-^10^BPA delivery. However, the challenge of 4-L-^10^BPA accumulation in cancer cells is related to the fact that L-type amino acid transporters function as antiporters: reduction of the extracellular concentration of L-^10^BPA leads to an efflux of intracellular L-10BPA, accompanied by its replacement with a different extracellular substrate (e.g., tyrosine) [11]. This mechanism impedes attainment of the intratumoral boron concentrations (20–50 μg ^10^B/g tumor, ~10^9^ atoms ^10^B/cell) needed for effective BNCT [12].

The clinical application of 4-L-^10^BPA is also significantly complicated by its poor solubility (0.6 g/L) in solutions with a neutral pH. Mori et al. proposed to use a complex of 4-L-^10^BPA with monosaccharides to enhance the solubility of 4-L-^10^BPA [13]. The usual method in the clinical application of 4-L-^10^BPA in BNCT involves intravenous administration of a 4-L-^10^BPA complex with *D*-fructose or sorbitol. However, this approach is far from ideal: thus, a patient weighing 60 kg systemically receives 1 L of a solution containing 30 g of 4-L-^10^BPA and 31.5 g of *D*-sorbitol. Such a high burden causes side effects such as hypoglycemia, hepatic, and renal failure in individuals with hereditary fructose intolerance (as a result of sorbitol metabolism), as well as hematuria resulting from 4-L-^10^BPA crystallization in urine [4, 14, 15].

Hence, designing novel formulations of boron-containing compounds that would improve drug accumulation in tumors and alleviate side effects is a top priority in fundamental medical research under the development of BNCT.

Liposomes are viewed as effective drug delivery systems owing to their lack of inherent toxicity, capacity to encapsulate large quantities of a drug in both aqueous and hydrophobic phases, as well as the potential for modifying the outer surface with ligands specific to tumor-associated antigens for active targeting [16].

Previously, we had developed a system for engineering nanoliposome (~ 100 nm) whose outer surface is modified with a module specifically targeting the human epidermal growth factor receptor 2 (HER2), while the inner aqueous environment contains a large quantity (up to 10,000 molecules per liposome) of protein toxins [17, 18, 19] or peptide nucleic acids [20]. This approach has been used in our study to engineer HER2-specific liposomes loaded with 4-L-^10^BPA.

## EXPERIMENTAL PART

**Engineering DARPin-modified liposomes loaded with 4-L-^10^BPA**

Accurately weighed samples of 4-L-^10^BPA (Katchem, Czech Republic), 10 and 15 mg (three replicates used for each weight), were dissolved in 300 μL of Milli-Q water and mixed with a *D*-fructose solution (Sigma, USA) at a 1 : 1 molar ratio. Next, 1 M NaOH was added slowly, dropwise (within 10–15 min) until complete dissolution of 4-L-^10^BPA had been achieved; pH of the solution was 10–10.5. Next, pH was slowly adjusted to 8.0 using concentrated 1 M HCl. A mixture of phospholipids prepared from granules of L-α- phosphatidylcholine (40%), phosphatidylethanolamine (16%), and phosphatidylinositol (11%) (Avanti Polar Lipids) was added to the resulting solution to a final concentration of 4 g/L. The mixture was subjected to five quick freeze–thaw cycles and extruded through a filter with a pore diameter of 100 nm. In order to remove the BPA–*D*-fructose complex not incorporated into the liposomes, the liposome mixture was passed through a NAP-5 column equilibrated with a 100 mM NaPi buffer, pH 8.0. Next, the liposomes were modified with 2-iminothiolane (Merck, Germany) for inserting the SH groups and the SH-containing liposomes were conjugated to DARPin_9-29 modified with a sulfo-EMCS heterobifunctional crosslinker (Thermo Fisher Scientific, USA), according to the protocol described in ref. [17].

The hydrodynamic size and ζ-potential of the targeted and non-targeted boron-loaded liposomes were determined on a Zetasizer Nano ZS analyzer (Malvern Instruments, UK). Prior to measurements, the samples in the solution containing 150 mM NaCl and 20 mM NaPi, pH 7.5, were diluted with water 25-fold. The ζ-potential values were calculated using the Smoluchowski approximation.

To be used in confocal microscopy and flow cytometry experiments, the DARP-Lip(BPA) samples were conjugated to AF-488 hydroxysuccinimide ester (Lumiprobe, Russia), according to the manufacturer’s protocol.

**Quantification of boron in liposomes**

The content of 4-L-^10^BPA loaded into the targeted liposomes was determined using a NexION 2000 inductively coupled plasma mass spectrometer (PerkinElmer). For this purpose, 50 μL of the liposome sample was dissolved in 300 μL of aqua regia, incubated at 70°C for 1 h, mixed with 1,200 μL of Milli-Q water, and analyzed by inductively coupled plasma mass spectrometry (ICP-MS). The liposome concentration was determined spectrophotometrically according to the procedure described in ref. [17] by recording the absorption spectrum in a quartz cuvette on an Ultrospec 7000 spectrophotometer (GE) in the wavelength range of 210–800 nm.

**Cell cultures**

Human ovarian carcinoma (SKOV-3) and cervical carcinoma (HeLa) cell lines were used in the study. Cells were cultured at 37°C in humidified atmosphere in a RPMI 1640 medium (PanEco, Russia) supplemented with 2 mM L-glutamine (PanEco), 10% fetal bovine serum (Gibco, USA), and antibiotics (10 U/mL penicillin, 10 μg/mL streptomycin, PanEco).

**Flow cytometry**

In order to assess the ability of DARP-Lip(BPA) to bind to HER2, 200,000 SKOV-3 and HeLa cells were incubated in a complete growth medium at 37°C for 10 min in the presence of DARP-Lip(BPA)-AF488 at different concentrations (350 or 150 nM) (concentration calculated on the basis of the dye; the concentration of the DARPin-modified liposomes was 1 nM and 0.5 nM, respectively). After the incubation, the cells were washed thrice with PBS and analyzed using a NovoCyte 3000 flow cytometer. The fluorescence of AF488 was excited using a 488-nm laser; fluorescence was detected in the 530 ± 30 nm channel (the FITC channel).

**Confocal microscopy**

Binding of the targeted module within DARP-Lip(BPA) to HER2 on the surface of SKOV-3 cells typically characterized by overexpression of this receptor was studied by confocal microscopy. Approximately 3,500 SKOV-3 cells were seeded into the wells of a 96-well glass-bottom microplate (Eppendorf) and cultured overnight. The next day, 250 nM of a DARP-Lip(BPA)-AF488 conjugate (concentration calculated on the basis of the dye) was added to the cells and the mixture was incubated for 20 or 120 min. The nuclei were stained with 10 nM Hoechst 33342 (10 min at 37°C). After washing of the cells thrice with PBS and addition of the FluoroBright medium (Gibco), an analysis using an LSM 980 confocal microscope (Carl Zeiss, Germany) with a 63× Plan-Apochromat oil-immersion objective was conducted. The fluorescence of Hoechst 33342 was excited using a 405-nm laser and detected at 410–520 nm; the fluorescence of AF488 was excited with a 488-nm laser and detected at 497–562 nm.

## RESULTS AND DISCUSSION

**Engineering and characterization of HER2- specific liposomes loaded with 4-L-^10^BPA**

The poor water solubility of 4-L-^10^BPA, low accumulation in tumor tissue, and rapid clearance are the main obstacles in the application of 4-L-^10^BPA for BNCT. Various 4-L-^10^BPA carriers that would enhance the compatibility of this compound with aqueous media, increase its accumulation in the target tissue, and extend its circulation time in the bloodstream are currently under development in the attempt to solve these problems [21, 22]. Liposomes 100–200 nm in diameter are the most commonly used drug delivery systems, since they penetrate through the fenestrated endothelium of blood vessel walls in tumors and can be accumulated in the underlying tumor tissue [23].

The tumor-associated antigen HER2, whose expression is typically upregulated in many human epithelial cancers [24], was selected as a target in liposomal targeting of cancer cells. In modern medical practice, the HER2 tumor marker is a therapeutic target for monoclonal antibodies (pertuzumab and trastuzumab) and kinase inhibitors (lapatinib) in patients with HER2- positive breast cancer [24].

The scaffold-designed ankyrin repeat protein DARPin_9-29 was used as a vector molecule to target nanoliposomes to a specific tumor-associated antigen. DARPin_9-29 is an antibody mimetic capable of highly specifically interacting with HER2 subdomain I (*K*D= 3.8 nM) [25].

**Fig. 1:**
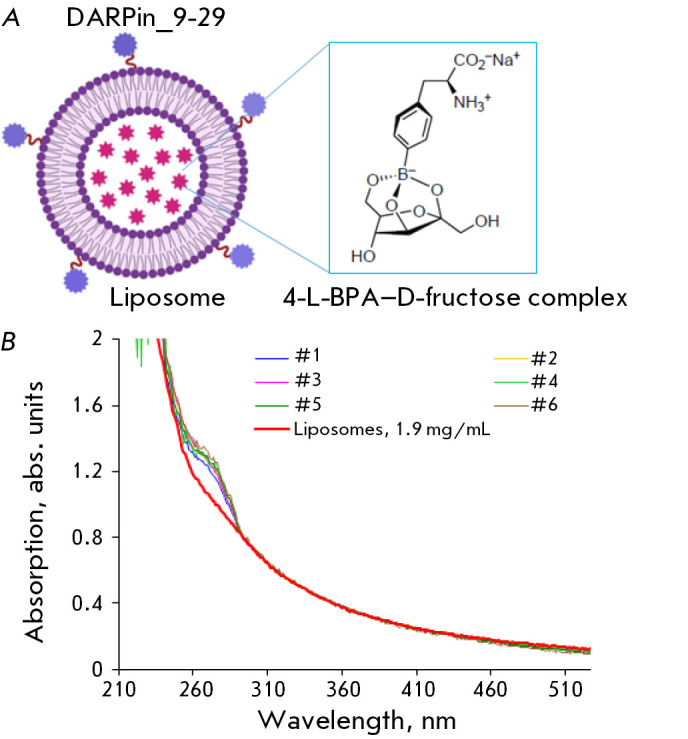
DARPin-modified liposomes loaded with the 4-L-^10^BPA-D–fructose complex. (A) The schematic representation of DARP-Lip(BPA). The internal environment of liposomes is loaded with the 4-L-^10^BPA-D–fructose complex. The outer surface is modified by the HER2-specific scaffold protein DARPin_9-29. (B) The absorption spectra of DARPin-modified samples #1–6 containing 4-L-^10^BPA and the absorption spectrum of empty liposomes with a concentration of 1.9 mg/mL (red curve)

4-L-^10^BPA was loaded into liposomes as part of a complex with *D*-fructose at a 1 : 1 molar ratio (*Fig. 1A*). To verify the reproducibility of the procedure of loading 4-L-^10^BPA into liposomes, six samples of liposomes loaded with 4-L-^10^BPA were prepared, starting at the stage of weighing of the 4-L-^10^BPA samples.

The absorption spectra of the samples #1–6 of DARPin-modified liposomes loaded with 4-L-^10^BPA have a characteristic peak at 270 nm due to the absorption of the incorporated 10BPA. The absorption spectra of empty liposomes obtained from a suspension of 1.9 mg/mL of phospholipids (the red curve in *Fig. 1B*) do not contain any peak at 270 nm. Otherwise, the spectra of empty and loaded liposomes are identical. We had previously determined that the molar concentration of a 1 mg/mL suspension of unmodified liposomes is 1.1 nM [17]. DARPin_9–29 is characterized by weak absorption at 280 nm, since the protein molecule has a very low content of aromatic residues (five phenylalanine and no tryptophan residues). Therefore, the presence of DARPin on the liposome surface does not alter the absorption spectrum of the liposome. Hence, the molar concentration of the liposomes in samples #1–6 is 2.09 nM.

Boron content in the liposomes was quantified by ICP-MS. Boron concentration in the liposome samples proved independent of the initial weighed sample (which might indicate that the degree of filling of the aqueous phase with the BPA–*D*-fructose complex in the liposome is at its maximum), on average equal to 258 ± 44 μM and corresponding to (1.2 ± 0.2) × 10^5^ 4-L-^10^BPA molecules per liposome.

**Fig. 2:**
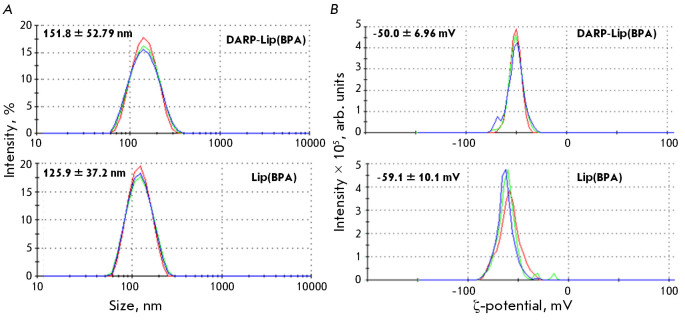
of the hydrodynamic size and ζ-potential of DARPin_9-29-modified and unmodified liposomes loaded with 4-L-^10^BPA. (A) The hydrodynamic size of DARP-Lip(BPA) and Lip(BPA). (B) The ζ-potential of DARP-Lip(BPA) and Lip(BPA)

The size and ζ-potential of 4L-10BPA-loaded liposomes, modified and non-modified with DARPin_9-29, were measured by dynamic and electrophoretic light scattering. Conjugation of liposomes and DARPin_9-29 increases their hydrodynamic diameter from 125.9 ± 37.2 to 151.80 ± 52.79 nm (*Fig. 2A*) and shifts the ζ-potential from -59.1 ± 10.1 to -50.0 ± 6.96 mV (*Fig. 2B*). The negative ζ-potential of the liposomes indicates that the sample is stable and not prone to aggregation.

**Analysis of the interaction specificity of DARP-Lip(BPA) with the HER2 receptor *in vitro***

**Fig. 3:**
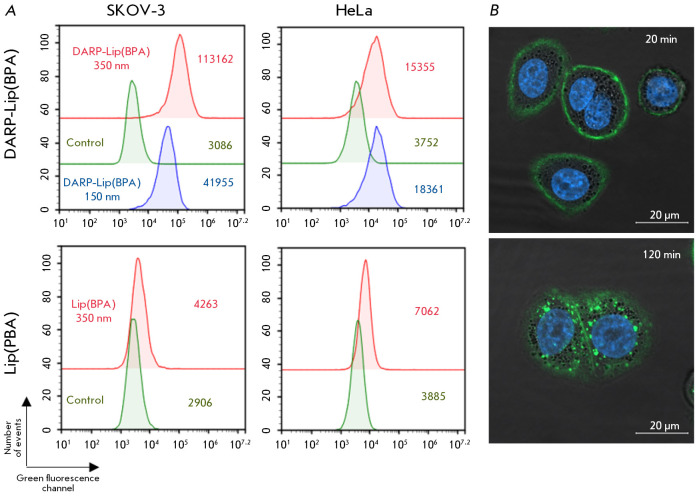
Interaction of DARP-Lip(BPA) with the HER2 receptor in vitro. (A) Evaluation of the specific interaction of DARP-Lip(BPA) (upper pictograms) and Lip(BPA) (lower pictograms) with HER2-positive SKOV-3 cells and HeLa cells with normal HER2 expression levels by flow cytometry. The mean fluorescence intensity in the green channel is indicated on the pictograms. The green curve corresponds to fluorescent-unlabeled cells (control). The blue and red curves correspond to cells treated with 150 nM and 350 nM of DARP-Lip(BPA), respectively. (B) Confocal microscopy study of the interaction between DARP-Lip(BPA) and SKOV-3 cells. The duration of incubation of the cells in the presence of DARP-Lip(BPA) is indicated. Nuclei are stained with Hoechst 33342

The ability of the targeted DARPin_9-29 module residing on the surface of 4L-10BPA-loaded liposomes to interact with HER2 on the cell surface was studied using two independent techniques: flow cytometry and confocal microscopy (*Fig. 3*). Since DARP-Lip(BPA) does not exhibit autofluorescence, the liposomes were conjugated to AF-488-NH fluorescent dye prior to their application in the aforementioned optical analysis methods. Two human cancer cell lines were used in the experiment: the SKOV-3 ovarian carcinoma cell line, characterized by an elevated HER2 level on the cell surface (106 receptors per cell), and the HeLa cervical carcinoma cell line, characterized by the normal (for all epithelial tissues) HER2 level (104 receptors per cell). The SKOV-3 and HeLa cells were incubated with DARP-Lip(BPA)/ AF488 at two concentrations: 150 and 350 nM, as described in the Experimental section. The flow cytometry data demonstrate that the interaction between DARP-Lip(BPA) and the cells was HER2-specific. Hence, for the HER2-overexpressing SKOV-3 cells, a higher DARP-Lip(BPA)/AF488 concentration in the cell suspension increased the shift of the fluorescence intensity with respect to the control (a green curve): ~ 13.6-fold for a DARP-Lip(BPA)/AF488 concentration of 150 nM (the blue curve) and ~ 36.9-fold for a DARP-Lip(BPA)/AF488 concentration of 350 nm (the red curve) (*Fig. 3A*, upper left pictogram). Meanwhile, the fluorescence intensity of HeLa cells proved to be virtually independent of the DARP-Lip(BPA)/AF488 concentration in the medium and differed from the control (the green line) four- and fivefold for 150 nM (the blue curve) and 350 nM (the red curve) DARP-Lip(BPA)/AF488, respectively (*Fig. 3A*, upper right pictogram). The reason behind this is the absence of unbound HER2 receptors accessible for interaction with DARP-Lip(BPA) on the HeLa cell surface. Non-targeting liposomes loaded with 4-L-^10^BPA shift fluorescence intensity in neither SKOV-3 nor HeLa cells, thus attesting to DARPin-mediated interaction between liposomes and cells (*Fig. 3A,* the lower series of pictograms).

The specificity of DARP-Lip(BPA) binding to HER2 on the surface of cancer cells was also confirmed by confocal microscopy. Thus, characteristic staining of the cell membrane was observed after the SKOV-3 cells had been co-incubated with DARP-Lip(BPA)/ AF-488 for 20 min (*Fig. 3B*, upper pictogram). Further incubation of cells in the presence of DARP-Lip(BPA) resulted in internalization of liposomes (during 120 min), as indicated by green pixels in the cytoplasm (*Fig. 3B*, lower pictogram).

## CONCLUSIONS

A total of ~ 10^9^^10^B atoms need to accumulate in a cancer cell in order to ensure effective BNCT [12]. The applicability of 4-L-^10^BPA in BNCT is constrained by its poor water solubility and low accumulation in cells. This study has proposed a method for engineering nanosized HER2-specific liposomes whose inner environment contains large quantities (~120,000 molecules per liposome) of 4-L-^10^BPA. *In vitro *studies demonstrated that the engineered liposomes effectively interacted with the HER2 receptor on the surface of cancer cells and were efficiently internalized. We believe that the ability of DARPin-modified liposomes to precision-deliver large quantities of 4-L-^10^BPA into cancer cells will help solve the problem of low 4-L-^10^BPA accumulation and possibly open up new avenues for BNCT.

## Abbreviations

BNRT: boron neutron capture therapy
HER2: human epidermal growth factor receptor II
4-L-10BPA: 10B-4-L-boronophenylalanine
